# Leisure sedentary time and elevated blood pressure: evidence from the statutory retirement policy

**DOI:** 10.3389/fpubh.2024.1468221

**Published:** 2024-10-18

**Authors:** Hao Li, Weihong Zeng

**Affiliations:** ^1^Jinhe Center for Economic Research, Xi’an Jiaotong University, Xi’an, Shaanxi, China; ^2^Center for Aging Health Research, Xi’an Jiaotong University, Xi’an, Shaanxi, China

**Keywords:** risky health behaviors, leisure sedentary time, diastolic blood pressure, systolic blood pressure, statutory retirement policy, China

## Abstract

**Objectives:**

The relationship between sedentary behaviors and elevated blood pressure remains inconclusive, and the socioeconomic mechanisms underlying the linkage are rarely discussed. Since retirement is often associated with behavioral changes that impact health, this study aims to provide evidence on changes in leisure sedentary time after the statutory retirement age on elevated blood pressure, along with the socioeconomic mechanisms.

**Methods:**

We utilized data from five waves (2004–2015) of the China Health and Nutrition Survey (CHNS), focusing on males aged 55–65 employed in the formal sector. Leisure sedentary time, the independent variable, was measured based on self-reported data, while diastolic (DBP) and systolic (SBP) blood pressure were the dependent variables. Using statutory retirement policy as an exogenous variation, we employed a continuous difference-in-differences (DID) framework and a propensity score matching difference-in-differences (PSM-DID) approach to examine the relationship between changes in leisure sedentary time after the statutory retirement age and elevated blood pressure. The analysis was conducted using ordinary least squares (OLS). To address potential endogeneity, we applied the instrumental variable (IV) method via two-stage least squares (2SLS).

**Results:**

Our findings indicate an increase in diastolic blood pressure after statutory retirement, attributed to increased leisure sedentary time. However, there was no significant increase in systolic blood pressure. Moreover, physical activity did not appear to offset this rise in blood pressure, while higher educational attainment and having family members employed in the medical field helped mitigate its negative effects.

**Conclusion:**

This study highlights the potential adverse impact of increased leisure sedentary time on diastolic blood pressure among middle-aged men in the formal sector, while also exploring the socioeconomic factors that may alleviate these effects. These results provide a foundation for public health initiatives aimed at addressing the rising prevalence of sedentary behavior and its association with blood pressure issues.

## 1 Introduction

Elevated blood pressure is a leading modifiable risk factor of numerous health risks and is associated with 10.8 million deaths per year worldwide (1). In China, the prevalence of elevated blood pressure has risen significantly, now affecting approximately a quarter of all adults and about half of older adults (2, 3). It is widely recognized that elevated blood pressure is related to age, with the highest incidence occurring among older adults (4).

As the population ages, the baby boomers born in the 1950s and 1960s in China are gradually retiring. Retirement marks a significant turning point in an individual’s life (5), signifying the end of employment and the onset of aging. This transition prompts people to re-plan their lives, potentially altering their behaviors and impacting their health. On the one hand, retirement can negatively affect health, as some researchers have found associations with reduced physical activity, increased sedentary behaviors (e.g., TV watching), more frequent insomnia, and increased smoking and drinking (6). On the other hand, retirement can also positively impact health through increased exercise, sufficient sleep, and healthier meal preparation (7).

The etiology of elevated blood pressure is multifaceted, influenced by both genetic predispositions and lifestyle factors. Besides age, lifestyle changes such as smoking (8), alcohol consumption (9), and dietary imbalances (10) are primary contributors. However, the relationship between sedentary behavior and elevated blood pressure is often overlooked. Adults spend an alarming 9–10 h/day sedentary (11). Accordingly, the phrase “sitting is the new smoking” has been coined by popular press to describe a current epidemic of many nations (12). Sedentary behavior increased with age, and older adults are those who spend more time on sitting (13). Retirement means more leisure time; increased leisure time post-retirement may lead to more unhealthy behaviors such as sedentary activities, especially watching TV (14). However, studies on the association between sedentary behaviors and elevated blood pressure have reported mixed findings, which highlight the limitations of previous studies, especially confounding factors and measurement error (15). For example, one study found no significant link between sedentary behaviors and elevated blood pressure (16), while others, such as Guo et al. and Chauntry et al., established connections between total sedentary behavior and elevated blood pressure (17, 18).

Given the limitations of previous studies, this paper utilizes the statutory retirement policy as an exogenous variation to examine the effect of changes in leisure sedentary time (e.g., TV watching) before and after the statutory retirement age on elevated blood pressure using a continuous difference-in-differences (DID) framework combined with propensity score matching difference-in-differences (PSM-DID) approach. Additionally, to address concerns about the endogeneity, we use instrumental variable (IV) method. We also conduct several checks to ensure the validity of our method, including sample considerations, parallel trend tests, placebo tests, and robustness checks.

The statutory retirement age in China was established in the 1950s, mandating retirement in the formal sectors such as government, public services, state-owned, and collectively owned enterprises. The retirement age is set at 60 for men, 50 for female workers, and 55 for female cadres. This study focuses on men in the formal sector as research shows that many women opt for early retirement before reaching the standard retirement age (19), while men typically retire at the mandatory age of 60. One study observed a significant increase in the proportion of retired males at age 60, but not among females at ages 50 or 55 (20). Additionally, structural adjustments in state-owned and collectively owned enterprises from the mid-1990s altered urban employment patterns, particularly for women. Women over 40 experienced a sharp decline in labor force participation, with many reporting themselves as retired or permanently withdrawn from the workforce (19). For this reason, including women in this study would not provide reliable conclusions, so we limit our analysis to men in the formal sector (20–23). Moreover, to accurately assess the influence on elevated blood pressure, we focus on males aged 55–65 as elevated blood pressure is generally believed to be related to age, encompassing 5 years before and after the statutory retirement age (21, 24).

This study contributes to the literature in several ways. First, by employing a continuous DID design, PSM-DID approach, and IV method, we offer new evidence on the relationship between leisure sedentary time and elevated blood pressure before and after retirement, filling gaps in the current literature. Second, while most existing studies focus on risk factors such as smoking, drinking, and diet, our study emphasizes the impact of sedentary behavior on blood pressure, providing new insights into this underexplored area.

## 2 Literature review and hypothesis development

In this section, we review and summarize existing research from two perspectives: the relationships among retirement, sedentary behaviors, and elevated blood pressure, as well as the socioeconomic mechanisms.

Research suggests that retirement consists of three stages: the near-retirement phase, the transition period, and the retirement stability period (25). In most countries, the legal retirement age is fixed, making retirement life predictable. During the pre-retirement stage, workers have expectations or concerns about retirement and make preparations and psychological adjustments for the transition (26). The transition period includes the first few years of retirement. Continuity theory implies that the lifestyle during this period is consistent with the pre-retirement stage, as it takes time to form new habits (27). For example, a study found a greater increase in television viewing time after retirement among those who had less physically demanding jobs (28). Additionally, increased leisure time post-retirement may lead to more risky behaviors such as smoking, drinking, or sedentary activities. Several studies have found that sedentary time increases during the transition period (29–31), especially television viewing (28, 32, 33). For example, one study found that total sedentary time increased by 73 min per day during the transition period, with TV viewing time increasing by 28 min per day (30).

Sedentary behaviors are associated with modern lifestyles and lead to many adverse health outcomes, including elevated blood pressure. Previous literature has documented the correlation between high levels of sedentary time and elevated blood pressure (34). Contemporary evidence shows a strong association between occupational sedentary behavior and elevated blood pressure (35). Additionally, high levels of leisure sedentary time have been linked to elevated blood pressure (36). For instance, individuals who spent more than 3 h per day watching television had a significantly higher risk of elevated blood pressure compared to those who watched television for 0–1 h per day (37). Blood pressure is categorized into diastolic and systolic blood pressure. Some studies have found that increased sedentary time is associated with higher diastolic blood pressure (18, 38), with limited association to systolic blood pressure (39). Therefore, we propose the following hypothesis:

Hypothesis 1: Prolonged sedentary time after retirement is associated with higher diastolic blood pressure but not with higher systolic blood pressure.

Research on the underlying mechanisms is limited and mainly focuses on biological dimensions. For example, watching television often involves prolonged periods of uninterrupted sitting, especially after dinner (40). This increases the risk of elevated blood glucose and triglyceride levels (41). High blood sugar leads to chronic inflammation and oxidative stress, further contributing to elevated blood pressure (42). However, biological mechanisms limit the possibility of intervention. From a socioeconomic perspective, some literature has documented the moderating effect of physical activity on the association between sedentary behaviors and blood pressure (43), whereas most studies distinguish between sedentary behaviors and inactivity, suggesting that physical activity is unlikely to offset the adverse effects of sedentary behaviors on blood pressure changes (44, 45). For example, one person may engage in the recommended amount of physical activity every day (not physically inactive) but still spend a lot of time sitting. Conversely, a person may sit very little but not reach the recommended level of physical activity (physically inactive). Therefore, we propose the following hypothesis:

Hypothesis 2a: Physical activity is unlikely to offset the adverse effect of sedentary behaviors on elevated blood pressure.

Socioeconomic status (SES), traditionally measured through levels of education, income, and occupation, is considered the most fundamental cause of health disparities (46). Although SES indicators are powerful determinants of health, they do not impact health directly but serve as proxies for other determinants (47). Educational attainment is the most important dimension. People with higher levels of education generally have better access to and understanding of health information and adopt healthier lifestyles to promote their health. For example, more education is associated with an improved diet, moderate alcohol consumption, and less sedentary behaviors (47, 48). Additionally, a good education enhances self-care capabilities and increases the likelihood of accessing higher-quality healthcare. Higher educational attainment also leads to better job opportunities, higher wages, and better living standards, facilitating superior healthcare access (49).

Furthermore, health literacy, a key channel linking SES and health outcomes, relates to individuals’ knowledge, motivation, and competencies to access, understand, appraise, and apply health information, and to make appropriate decisions relevant to health promotion, disease prevention, and self-care management (50). Individuals with inadequate health literacy are more likely to report a sedentary lifestyle (51) and elevated blood pressure (52). Medical professionals, with higher health literacy, can provide more effective medical advice to promote health for both their patients and family members. Therefore, we propose the following hypothesis:

Hypothesis 2b: Educational attainment and having family members employed as medical workers are mechanisms through which prolonged sedentary time impacts elevated blood pressure.

## 3 Data discerption and empirical strategy

### 3.1 Data

The data used in this paper come from the China Health and Nutrition Survey (CHNS). The CHNS is an ongoing large-scale study aimed at determining how China’s social and economic development affects the health and nutritional status of the country’s population, with 10 rounds of collected data from 1989 to 2015 (i.e., 1989, 1991, 1993, 1997, 2000, 2004, 2006, 2009, 2011, and 2015). Jointly collected by the Chinese Center for Diseases Control and Prevention and the University of North Carolina at Chapel Hill, the CHNS survey randomly selected samples from 12 provinces, covering 12,522–20,878 individuals in each round.

### 3.2 Variables

#### 3.2.1 Blood pressure

We utilize diastolic blood pressure (DBP) and systolic blood pressure (SBP) as main dependent variables. The CHNS dataset encompasses diastolic blood pressure and systolic blood pressure measurements conducted by professionally trained medical staff for each individual. Specifically, the medical staff measured both the DBP and SBP three times on three consecutive days for each individual (53). We calculate the average DBP and SBP as main dependent variables. In our robustness check, we use the average DBP and SBP from the last two measurements instead of all three, considering that the first measurement might be more susceptible to measurement errors and less reflective of the individual’s “true” underlying blood pressure due to stress or anxiety (54).

#### 3.2.2 Leisure sedentary time

While TV watching is often the most common sedentary activity, sedentary behavior includes various activities, and measuring only TV watching may underestimate total sedentary time. In our study, leisure sedentary behavior was assessed through several questions. Participants were asked, “Do you engage in these sedentary activities?” The listed activities included watching TV, watching videos, and playing video games. Additional questions were asked to determine the time spent on these activities: “How much time do you spend on these activities from Monday to Friday?” and “How much time do you spend on Saturday and Sunday?” Based on these responses, we calculate total leisure sedentary time. Using non-exercise sitting behaviors, especially screen-based activities, is common in research. These measures have demonstrated acceptable reliability and validity (55), and have been widely employed in prior studies (56, 57).

We calculate the independent variable based on equation 1 and winsorize at the 1st and 99th percentiles of their respective sample distributions. The equation is as follows:


(1) xit=xweekdayit∗57+xweekendit∗27

According to dependent variable and independent variable, we employ the CHNS from the 2004, 2006, 2009, 2011, and 2015 waves.

#### 3.2.3 Control variables

In our specification, we control for a rich set of individual-, household-, and macro-level characteristics. First, we include individual and household factors such as sleep duration, physical activity, use of antihypertensive drugs, body mass index (BMI), smoking status, drinking status, marital status, and log-transformed annual household income. Second, as diet is linked to elevated blood pressure, we account for key nutritional elements, including average daily intake of dietary fat, carbohydrates, and protein (g/d) (58). We also control for taste preferences (e.g., a preference for salty food) and attitudes toward the relationship between salt intake and blood pressure to reduce omitted variable bias. A strong preference for salty food, combined with the belief that salt does not affect blood pressure, suggests a high-salt diet. Third, we control for mental health and other chronic diseases such as diabetes as they are associated with elevated blood pressure (59, 60). Mental health was assessed using the CHNS dataset, which includes three questions for individuals aged 55 and older: “Do you have as much energy as you did last year?” “Are you as happy now as you were when you were younger?” and “As you age, are things better than you expected?” Each question has five possible responses, scored from 1 to 5. A higher score indicates greater agreement with the statements, and the total score was used as a mental health measure. These questions are simplified versions of indicators from the Symptom Checklist-90 (SCL-90), reflecting the mental health of individuals (61). In addition, blood pressure status is strong associated with elevated blood pressure. Hypertensive population have greater variability in blood pressure than normotensive population (62), especially taking into account sedentary behaviors (63). We also control for occupational sedentary time, as it significantly influences blood pressure (64). Furthermore, we include macro-level variables such as Urbanization, Economic, Health, and House Scores, where higher scores indicate better development. We also account for individual, household, and year fixed effects to control for confounding factors, with age effects absorbed by the fixed effects.

Genetic predisposition can also affect blood pressure (65). Since the CHNS dataset does not provide family health history, we mitigate potential omitted variable bias through three strategies: (1) controlling for individual fixed effects, which capture time-invariant factors such as genetics that could affect both sedentary time and blood pressure; (2) employing an instrumental variable (IV) method; and (3) excluding individuals and regions with a strong preference for salty foods in our robustness checks, as taste preferences tend to be stable and are correlated with elevated blood pressure.

Table 1 presents descriptive statistics for the sample. The average diastolic blood pressure is 83 mmHg, while the average systolic blood pressure is 131 mmHg, indicating that most participants are normotensive. On average, participants engage in 154 min of leisure sedentary time per day and 1,026 min of occupational sedentary time per week before retirement. On average, participants sleep 7.76 h per day and have a mean BMI of 24, with values ranging from 17 to 32. Most people do not prefer salty food and believe that salty food raises blood pressure. The proportions of individuals who exercise, have diabetes, or take antihypertensive drugs are relatively low, with average values of 0.23, 0.08, and 0.20, respectively. About half of the sample smokes or drinks alcohol, and most are married. The average mental health score is 9, ranging from 3 to 15. The average daily intake of dietary fat, carbohydrates, and protein is 85.22 g, 280.73 g, and 74.84 g, respectively, similar to findings from a previous study (66). The average annual household income is 56,016 yuan.

**Table 1:** Variables definition and descriptive statistics.

	Definition (%)	Mean
Outcomes		
Diastolic blood pressure (DBP)	The average DBP for 3 days, mmHg	83
Systolic blood pressure (SBP)	The average SBP for 3 days, mmHg	131
Independent variable		
Leisure sedentary time	The average leisure sedentary time (min)	154
Covariates		
Sleep time	Sleep time (h)	7.67
Physical activity	0 = never workout (81); 1 = participate in one sport (16); 2 = participate in two different sports (2); 3 = participate in three different sports (1).	0.23
Taste preference	1 = strongly dislike salty food (17); 2 = dislike salty food (67); 3 = neutral (11); 4 = like salty food (4); 5 = very like salty food (1).	2.01
Taste attitude (the association between eating salty food and elevated blood pressure)	1 = strongly disagree (0); 2 = disagree (2); 3 = neutral (3); 4 = agree (92); 5 = strongly agree (3).	3.95
Body mass index (BMI)	Body mass index	24
Blood pressure status	0 = DBP < 90 and SBP < 140 (64); 1 = DBP ≥ 90 or SBP ≥ 140 (20); 2 = DBP ≥ 90 and SBP ≥ 140 (16).	0.52
Diabetes	0 = No (92); 1 = Yes (8).	0.008
Antihypertensive drugs	0 = No (79); 1 = Yes (21).	0.21
Average fat intake (g/d)	Average fat intake	85.22
Average carbohydrate intake (g/d)	Average carbohydrate intake	280.73
Average protein intake (g/d)	Average protein intake	74.84
Mental health	Mental health	9
Occupational sedentary time (ST)	The average weekly working time (min)	1,026
Smoke status	0 = No (41); 1 = Yes (59).	0.59
Alcohol status	0 = No (41); 1 = Yes (59).	0.59
Marital status	0 = Otherwise (4); 1 = Married (96).	0.96
Family income	Annual household income (RMB yuan)	56,016
Urbanization	Urbanization index	83
House scores	House scores	9
Economic scores	Economic scores	9
Health scores	Health scores	7

**Figure 1:**
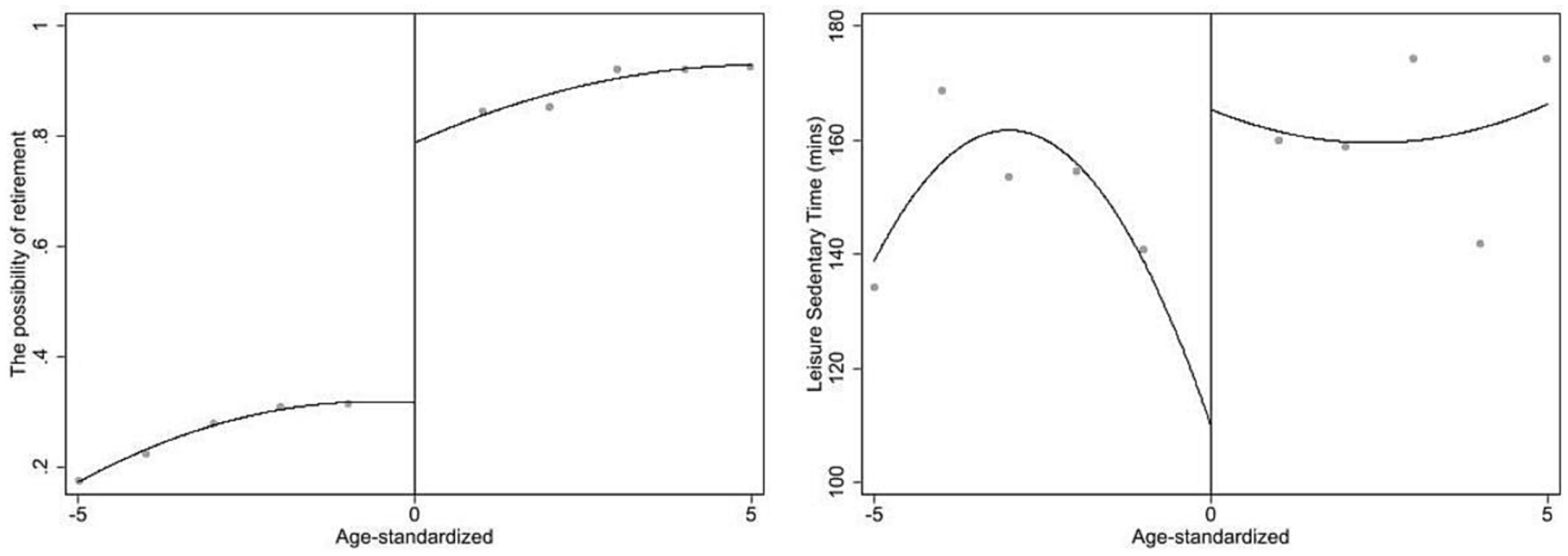
The possibility of retirement and leisure sedentary time before and after law-forced retirement policy.

**Figure 2:**
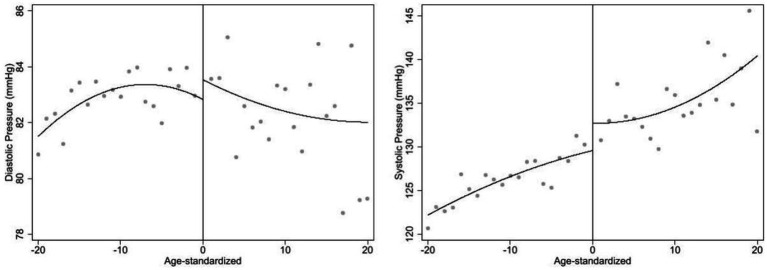
The changes of diastolic blood pressure and systolic blood pressure before and after law-forced retirement policy.

Additionally, we provide the evidence of the changes in retirement possibility and leisure sedentary time before and after retirement in Figure 1, and the changes in blood pressure before and after retirement in Figure 2. Figure 1 displays the relationships between the statutory retirement policy and retirement possibility on the left hand, and leisure sedentary time on the right hand. There is a clear positive discontinuity in both retirement possibility and leisure sedentary time before and after retirement. The fitted line in Figure 1 suggests that the discontinuity of retirement possibility is roughly 50 percentage points around the cut-off on the left hand, and the discontinuity of leisure sedentary time is roughly 40 min around the cut-off on the right hand. In sum, Figure 1 demonstrate that leisure sedentary time increases significantly after retirement.

Figure 2 shows changes in blood pressure before and after retirement. Diastolic blood pressure increases on the right, while systolic blood pressure shows a similar increase on the left. After retirement, diastolic blood pressure tends to decline with age, while systolic blood pressure continues to rise, consistent with previous studies that found diastolic blood pressure increases with age until about 55, then declines, while systolic blood pressure continues to rise at least until age 80 (67).

### 3.3 Empirical strategy

#### 3.3.1 DID framework

This paper uses the statutory retirement policy as an exogenous variation to examine the effect of changes in leisure sedentary time before and after retirement on elevated blood pressure under the continuous difference-in-differences (DID) framework, based on males aged between 55 and 65 who worked in the formal sector.

There are three common ways to define retirement (68): (1) self-reported retirement status. This is not ideal in this study because some people still work for pay after retirement. (2) The statutory retirement policy. This is not ideal because some people may manipulate their retirement status, such as those who are either unhealthy or senior cadres. The former group may retire early (69), while the latter group may delay retirement (70). Thus, we adopt the third definition using the statutory retirement policy as an exogenous variation and excluding the individuals who still work for pay after retirement, and who may manipulate their retirement status.

The regression framework of the continuous DID design is typical written as equation 2 as follows.


(2) $$Y_{\mathrm{it}}=\theta_{0}+\theta_{1}\mathrm{sedentar}y_{\mathrm{it}}*D_{\mathrm{it}}+\theta_{2}X_{*t}+\delta_{i}+\delta_{h}+\tau_{t}+\varepsilon_{\mathrm{it}}$$

Where $Y_{\mathrm{it}}$ is dependent variables (the average DBP and the average SBP) for individual i at wave t , and $\mathrm{sedentar}y_{\mathrm{it}}$ represents individual $i^{\prime }s$ leisure sedentary time at wave t . $D_{\mathrm{it}}$ is a dummy variable which equals 1 if the subject is from the treatment group, 0 if from the control group. $X_{*t}$ is a set of control variables, and $\delta_{i}$ , $\delta_{h}$ and $\tau_{t}$ represents individual fixed effect, household fixed effect, and year fixed effect. The coefficient of interest is $\theta_{1}$ . If retirees who spend more leisure time on sitting elevates blood pressure, we should expect $\theta_{1}>0$ .

#### 3.3.2 PSM-DID framework

DID estimation is most appropriate when the treatment is randomly assigned or at least when observable characteristics can be used to control for the treatment. Although the statutory retirement policy is an exogenous variation, the DID results may be biased by possible unobservable and unchangeable intergroup differences between the treatment and control groups. Therefore, a comparable control group is often constructed using matching techniques. Rosenbaum and Rubin suggest matching on the propensity score (PSM) (71). The PSM can find the most similar samples in the treatment group and the control group for comparison; after matching the samples, both groups do not differ significantly in the observable control variables. Therefore, we employ the propensity score matching difference-in-differences (PSM-DID) framework to further ensure our results’ robustness.

#### 3.3.3 Instrumental variable method

To further improve the validity of our results, we employ instrumental variable (IV) method to address the endogenous concern of leisure sedentary time. While the fixed effect model is generally known to be effective in addressing omitted variable bias, measurement error is main endogeneity in our study, because our independent variable was collected by questionnaires, which is inevitably underestimating leisure sedentary time (72). Therefore, $\hat{\beta_{1}}$ will underestimate $\beta_{1}$ if $\beta_{1}$ is positive.

To alleviate the endogeneity problem, this paper uses external instrumental variable method and higher moment instruments approach proposed by Lewbel for further regressions (73).

For the external instrumental variable method, we use “Do you like watching TV?” as an instrumental variable. Responses ranged from “dislike very much” as 1 to “like very much” as 5, with higher scores representing greater preference. An ideal instrumental variable must satisfy the following two conditions: relevance and exogeneity (exclusion restriction). In terms of relevance, screen use preference is strongly associated with screen use time. For example, a study in China showed that the screen preference was significantly positively associated with TV viewing time (74). As for exogeneity, screen use preference is a subjective attitude unrelated to health outcomes, satisfying the exclusion restriction.

Moreover, we adopt higher moment instruments approach proposed by Lewbel to construct an internal instrumental variable as the method without relying on external factors. Lewbel suggests using the cubic relationship between independent variables and the mean value of their higher moments. This approach, originally designed for measurement error models, has proven useful in dealing with general correlated-regressor errors and multilevel models. For example, one study examining the effects of TV viewing on children’s cognitive outcomes used Lewbel’s IV to correct for measurement error bias (75). Following this framework, we take the cubic between the individual’s leisure sedentary time and the mean value of their family’s leisure sedentary time as the instrumental variable. This construction is strongly correlated with leisure sedentary time but is highly unlikely to be correlated with elevated blood pressure, thus addressing endogeneity concerns (75).

#### 3.3.4 Moderating effect

Physical activity has been defined as “any bodily movement produced by skeletal muscles that results in energy expenditure (76).” It can be classified into three intensities: light (1.6–2.9 MET (metabolic equivalent of task), such as slow walking or household chores); moderate (3.0–6.0 MET, such as jogging, golfing, light cycling, or dancing); and vigorous (>6.0 MET, such as football, tennis, running, or boxing) (77). Based on these classifications, we explore the moderating effect of physical activity by examining both moderate physical activities (MPA), like walking, jogging, and dancing, and vigorous physical activities (VPA), like football, tennis, basketball, and badminton. Specifically, we use interaction terms between physical activity time and the independent variable to assess these moderating effects.

Socioeconomic status (SES) is defined as an individual’s or group’s position within a hierarchical social structure, reflecting social class and status (78). In this study, we focus on families with low educational attainment and family members who are not employed as medical workers for two key reasons. First, there are relatively few individuals in our dataset with high educational attainment or who work as medical professionals, which could lead to unstable results for this group (less than 5% of individuals are medical professionals). Second, these individuals likely have more accurate health perceptions, and focusing solely on them may yield insignificant findings. Instead, we aim to show that individuals with low educational attainment and families without medical workers experience more pronounced changes in blood pressure.

## 4 Empirical results

### 4.1 DID and PSM-DID framework

Table 2 presents the estimates of the effect of leisure sedentary time on elevated blood pressure. The first two columns use the continuous DID model, while the last two columns use the PSM-DID model. Columns (1) and (3) indicate significant positive effects of increased leisure sedentary time after retirement on diastolic blood pressure, passing the 1% significance tests. Specifically, the results show an average increase of 0.011 mmHg in diastolic blood pressure in column (1) and (3). A back-of-the-envelope calculation suggests that each additional hour of sedentary behavior per day significantly increase in diastolic blood pressure by 0.66 mmHg on average. Our work is similar to Lee and Wong, who found that each hour increase in self-reported sedentary behavior was associated with increase in diastolic blood pressure of 0.20 mmHg (79). In contrast, columns (2) and (4) show that increased leisure sedentary time is unlikely to affect systolic blood pressure after retirement. These findings support Hypothesis 1.

**Table 2:** The effect of leisure sedentary time on elevated blood pressure.

	Continuous DID		PSM-DID	
	DBP	SBP	DBP	SBP
Sedentary*D_it_	0.011^***^	−0.003	0.011^***^	−0.006
	(0.004)	(0.006)	(0.004)	(0.006)
Sleep time	0.311	−0.374	0.245	−0.545
	(0.311)	(0.463)	(0.323)	(0.495)
Physical activity	−0.006	−0.841	−0.419	−0.545
	(0.616)	(0.916)	(0.643)	(0.985)
Taste preference	0.472	0.327	0.373	0.251
	(0.512)	(0.762)	(0.559)	(0.855)
Taste attitude	2.143^*^	−3.489^*^	2.279^*^	−3.451^*^
	(1.193)	(1.775)	(1.195)	(1.830)
BMI	0.466^*^	1.089^***^	0.371	1.194^***^
	(0.256)	(0.381)	(0.260)	(0.399)
Blood pressure status	7.606^***^	10.834^***^	7.516^***^	10.702^***^
	(0.498)	(0.740)	(0.511)	(0.782)
Diabetes	−2.877	1.153	−1.543	1.328
	(1.752)	(2.607)	(1.830)	(2.802)
Antihypertensive drugs	−1.673^*^	−0.104	−1.208	−0.152
	(0.940)	(1.398)	(0.981)	(1.501)
Average fat intake	0.002	−0.011	0.005	−0.009
	(0.010)	(0.015)	(0.010)	(0.016)
Average carbohydrate intake	−0.006	0.002	−0.004	0.001
	(0.005)	(0.007)	(0.005)	(0.008)
Average protein intake	−0.009	0.033	−0.001	0.032
	(0.019)	(0.028)	(0.019)	(0.030)
Mental health	0.062	−0.125	0.098	−0.072
	(0.168)	(0.250)	(0.174)	(0.267)
Occupational ST	0.001^**^	0.001^**^	0.001	0.001
	(0.000)	(0.001)	(0.000)	(0.001)
Smoke status	1.160	0.192	1.030	−0.079
	(0.929)	(1.382)	(0.941)	(1.440)
Alcohol status	0.187	3.151^**^	−0.126	3.438^**^
	(0.868)	(1.292)	(0.901)	(1.379)
Marital status	0.380	4.308	−1.020	4.319
	(2.797)	(4.160)	(2.770)	(4.241)
Family income	−0.907^*^	−0.450	−0.774	−0.346
	(0.513)	(0.764)	(0.523)	(0.801)
Urbanization	0.006	0.214^**^	0.029	0.183^*^
	(0.065)	(0.097)	(0.068)	(0.104)
House scores	−0.772	0.244	−0.610	0.827
	(0.668)	(0.994)	(0.736)	(1.127)
Economic scores	0.128	−0.031	0.167	0.120
	(0.225)	(0.335)	(0.233)	(0.356)
Health scores	0.217	−0.463	0.203	−0.585^*^
	(0.191)	(0.285)	(0.198)	(0.303)
Individual FE	YES	YES	YES	YES
Household FE	YES	YES	YES	YES
Year FE	YES	YES	YES	YES
Constant	69.235^***^	95.094^***^	66.716^***^	89.535^***^
	(11.982)	(17.825)	(12.378)	(18.952)
Obs.	590	590	535	535
*R*^2^	0.811	0.842	0.819	0.847

Significance: *, ** and *** denote significance at 10, 5 and 1% level, respectively.

Additionally, two assumptions must be met when using the propensity score matching: the balance assumption and the common support assumption. The balance assumption requires that the matching variables balance the data well, meaning no significant difference exists between the treatment and control groups after matching. The common support assumption ensures sufficient overlap between the treatment and control group samples, allowing for adequate matching.

**Figure 3:**
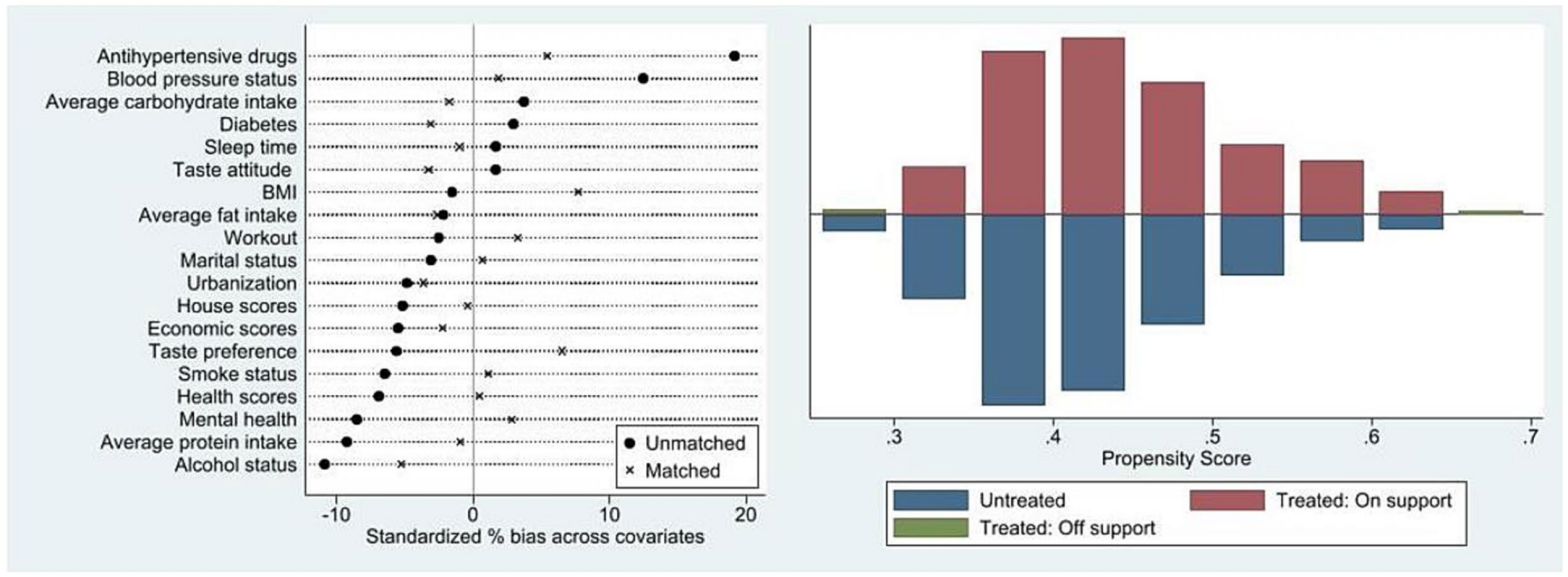
Results of the balance assumption test & results of the common assumption test.

Figure 3 provides the results of these assumption tests. The balance assumption test, shown on the left, indicates that standardized biases are largely reduced, with all selection biases below 10%, suggesting effective elimination of selection bias by matching. Our balance assumption test excludes occupational sedentary time and family income, as the former is zero after retirement and the latter significantly reduces after retirement. The common support assumption test, shown on the right, demonstrates that most samples fall within the common value range, indicating minimal sample loss after matching and that the common support assumption is met.

### 4.2 Instrumental variable method

Possible endogeneity may arise from measurement error bias. To alleviate this issue, this paper uses three additional checks to ensure the robustness, including external instrumental variable method (by using Preference for watching TV), higher moment instruments approach (by using Lewbel’s IV), and a combination of both methods.

Table 3 presents the results from the external instrumental variable method. Column (3) shows the first-stage IV estimates, indicating a significant positive relationship between the instrument (preference for watching TV) and the endogenous independent variable (leisure sedentary time). The first-stage F-test yields an F-statistic of 14.40, which exceeds the threshold for weak instruments, as suggested by Stock and Yogo (80). Additionally, the *p*-value of the Kleibergen-Paap rk LM statistic is 0.00, rejecting the null hypothesis and confirming the validity of the instrument. The second-stage results, shown in columns (1) and (2), demonstrate that increased leisure sedentary time after retirement has a significant positive effect on diastolic blood pressure (column 1). The effect on systolic blood pressure (column 2), while positive, is not statistically significant. Specially, the coefficient in column (1) is larger than those in columns (1) and (3) of Table 2, suggesting a potential downward bias in the estimates when endogeneity is not addressed. Then, the positive coefficient in column (2) implies that increased leisure sedentary time also affects systolic blood pressure after adjusting for endogeneity, consistent with the trend shown in Figure 2.

**Table 3:** Endogenous test of the effect of leisure sedentary time on elevated blood pressure (preference for watching TV).

	DBP	SBP	First stage	DBP	SBP
Sedentary*D_it_	0.012^*^	0.001		0.011^***^	−0.003
	(0.006)	(0.009)		(0.004)	(0.006)
IV			25.048^***^	0.030	−0.536
			(6.602)	(0.491)	(0.730)
Covariates	YES	YES	YES	YES	YES
Fixed-effect	YES	YES	YES	YES	YES
Constant	70.533^***^	93.340^***^	398.335^**^	69.169^***^	96.300^***^
	(12.065)	(17.852)	(162.271)	(12.051)	(17.913)
F-stat (First stage)			14.40		
Kleibergen-Paap rk LM stat			24.877^***^		
Obs.	590	590	590	590	590
*R*^2^	0.809	0.842	0.648	0.811	0.842

All models include fixed effects for individual, household, and year, plus all covariates.

To further validate the instrumental variable’s effectiveness, we conduct a reduced form regression, following the approach of Acemoglu et al. (81). Columns (4) and (5) include both the independent variable and the instrumental variable in the model. We find that the coefficient of IV is insignificant after controlling for the independent variable, which, to some extent, supports that the IV will not directly affect dependent variable in ways other than independent variable.

Similarly, Table 4 reports the results from the higher moment instruments approach, which align with the earlier findings. Table 5 combines the external instrumental variable method with the higher moment instruments approach, and the results remain consistent. Thus, our primary conclusion—that increased leisure sedentary time raises diastolic blood pressure—is robust after addressing endogeneity.

**Table 4:** Endogenous test of the effect of leisure sedentary time on elevated blood pressure (Lewbel’s IV).

	DBP	SBP	First stage	DBP	SBP
Sedentary*D_it_	0.013^**^	0.004		0.011^**^	−0.002
	(0.005)	(0.008)		(0.004)	(0.007)
Lewbel’s IV			0.000^***^	−0.000	−0.000
			(0.000)	(0.000)	(0.000)
Covariates	YES	YES	YES	YES	YES
Fixed-effect	YES	YES	YES	YES	YES
Constant	69.946^***^	92.504^***^	192.689^*^	69.169^***^	96.300^***^
	(11.990)	(17.805)	(113.706)	(12.051)	(17.913)
F-stat (First stage)			370.58		
Kleibergen-Paap rk LM stat			313.429^***^		
Obs.	590	590	590	590	590
*R*^2^	0.810	0.842	0.828	0.811	0.842

All models include fixed effects for individual, household, and year, plus all covariates.

**Table 5:** Endogenous test of the effect of leisure sedentary time on elevated blood pressure (preference for watching TV + Lewbel’s IV).

	DBP	SBP	First stage
Sedentary*D_it_	0.013^**^	0.002	
	(0.005)	(0.008)	
IV			17.551^***^
			(4.540)
Lewbel’s IV			0.000^***^
			(0.000)
Covariates	YES	YES	YES
Fixed-effect	YES	YES	YES
Constant	70.137^***^	92.941^***^	149.109
	(11.983)	(17.795)	(111.925)
F-stat (First stage)			200.66
Kleibergen-Paap rk LM stat			325.55^***^
Sargan test *p*-value			0.8341
Obs.	590	590	590
*R*^2^	0.810	0.842	0.835

All models include fixed effects for individual, household, and year, plus all covariates.

Moving to column (3) of Table 5, we provide the tests regarding relevance and exogeneity. The first stage estimates show that we reject the hypothesis of the instruments are weak. Concurrently, Sargan test *p*-values exceed 0.1, confirming the exogeneity of the instrumental variables. Hence, our instrumental variable selection is reasonable.

### 4.3 Validity of DID model

We then perform a series of checks to bolster the validity of our methods. First, we address potential concerns about sample composition. We limit our analysis to white-collar male workers, identified as those in senior professional/technical, junior professional/technical, administrative/executive/managerial, or office staff roles. We also exclude individuals living in rural areas, as urban statutory retirement policies are more stringent. The first two columns of Table 6 present results for white-collar male workers, while the last two columns show results for individuals living in urban areas. Our findings remain consistent with our expectations.

**Table 6:** DID results-address the sample issue.

	White-collar workers		Urban workers	
	DBP	SBP	DBP	SBP
Sedentary*D_it_	0.010^**^	0.000	0.009^**^	−0.004
	(0.005)	(0.007)	(0.005)	(0.007)
Covariates	YES	YES	YES	YES
Fixed-effect	YES	YES	YES	YES
Constant	61.110^***^	93.551^***^	66.544^***^	121.748^***^
	(14.352)	(20.325)	(16.960)	(25.340)
Obs.	467	467	362	362
*R*^2^	0.796	0.845	0.841	0.861

All models include fixed effects for individual, household, and year, plus all covariates.

The identification assumption of the continuous DID method requires comparability between the treatment group and the control group. Therefore, to address a potential concern about the parallel trend assumption, we conduct an event study analysis and the empirical model (see Equation 3) used for this analysis is:


(3) $$y_{\mathrm{it}}=\rho_{0}+\rho_{1}\overset{65}{\underset{s=55}{\sum }}\mathrm{sedentar}y_{\mathrm{it}}*\mathrm{ag}e_{s}+\rho_{2}X_{*t}+\delta_{i}+\delta_{h}+\tau_{t}+\varepsilon_{\mathrm{it}}$$

**Figure 4:**
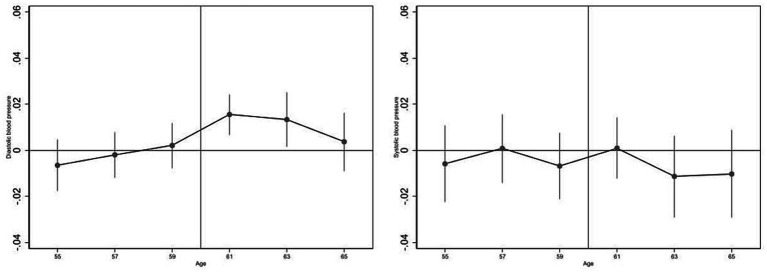
Event-study analyses of parallel trend tests.

The results, visualized in Figure 4, show that prolonged sedentary time during the transition period of retirement elevates diastolic blood pressure. However, there is no significant effect on systolic blood pressure changes before and after retirement, supporting the parallel trend assumption.

Finally, we examine our identification assumption using placebo tests. One placebo cut-off sets the retirement age for males in the formal sectors at 55 instead of 60, and another sets the retirement age for males in the informal sectors at 60. The results in Table 7 indicate no significant blood pressure changes from leisure sedentary time among “retirees,” specifically 55-year-old males in the formal sectors and 60-year-old males in the informal sectors.

**Table 7:** DID results-placebo tests.

	Retirement age of 55 in the formal sectors		Retirement age of 60 in the informal sectors	
	DBP	SBP	DBP	SBP
Sedentary*D_it_	−0.005	0.001	−0.003	−0.001
	(0.004)	(0.006)	(0.004)	(0.005)
Covariates	YES	YES	YES	YES
Fixed-effect	YES	YES	YES	YES
Constant	54.689^***^	95.886^***^	67.097^***^	93.195^***^
	(13.233)	(18.840)	(8.372)	(12.592)
Obs.	641	641	1,059	1,059
*R*^2^	0.797	0.830	0.818	0.841

All models include fixed effects for individual, household, and year, plus all covariates.

## 5 Moderating effect

We explore the mechanisms through which leisure sedentary time may lead to elevated blood pressure. First, we assess the role of physical activity. If increased physical activity effectively reduces elevated blood pressure, we would expect the interaction terms to be negative and statistically significant. However, Table 8 shows no significant moderating effect, supporting Hypothesis 2a. Our findings align with a systematic review that concluded sedentary behavior is not mediated by time spent in physical activity (82). Next, we examine the moderating effect of SES, focusing on individuals with low educational attainment and those whose family members do not work in the medical field. According to Hypothesis 2b, we predict that the effect on diastolic blood pressure in Table 9 will be greater than in Table 2. As expected, Table 9 demonstrates that educational attainment and support from medical professionals can mitigate elevated blood pressure. These findings support the view that SES is a “fundamental cause” of health (83).

**Table 8:** Moderating effect-physical activities as moderators.

	DBP	SBP	DBP	SBP
Sedentary*D_it_	0.011^***^	−0.004	0.011^***^	−0.004
	(0.004)	(0.006)	(0.004)	(0.006)
MPA	−0.012	0.022		
	(0.027)	(0.040)		
VPA			0.065	−0.027
			(0.083)	(0.123)
Interaction	−0.000	0.000	−0.000	0.001
	(0.000)	(0.000)	(0.001)	(0.001)
Covariates	YES	YES	YES	YES
Fixed-effect	YES	YES	YES	YES
Constant	70.088^***^	94.211^***^	68.742^***^	95.032^***^
	(12.068)	(17.957)	(12.024)	(17.876)
Obs.	590	590	590	590
*R*^2^	0.812	0.840	0.812	0.842

All models include fixed effects for individual, household, and year, plus all covariates.

**Table 9:** Moderating effect-SES as moderators.

	Individuals with low educational attainment		Family members not working as medical professionals	
	DBP	SBP	DBP	SBP
Sedentary*D_it_	0.020^***^	−0.005	0.013^***^	−0.004
	(0.007)	(0.011)	(0.004)	(0.006)
Covariates	YES	YES	YES	YES
Fixed-effect	YES	YES	YES	YES
Constant	70.851^***^	125.581^***^	66.870^***^	87.063^***^
	(19.301)	(31.355)	(12.354)	(18.475)
Obs.	255	255	562	562
*R*^2^	0.855	0.853	0.815	0.844

All models include fixed effects for individual, household, and year, plus all covariates.

## 6 Robustness checks

### 6.1 Subsample

One limitation of this paper is we lack the blood pressure information of agents’ family members, which may underestimate our result. To alleviate this concern, we rule out individuals who (1) live in Beijing and Shanghai (the highest hypertension prevalence provinces), and Guizhou (the lowest hypertension awareness province) (84), and Heilongjiang and Liaoning (traditional high salt intake provinces), and strongly like and like salty food; (2) are obesity (BMI over 28); (3) are diagnosed with diabetes. Table 10 reports the results of subsample. Panel A reports the findings for individuals living in the excluded provinces and those with a preference for non-salty food (first two columns). The last two columns of panel A report the results individuals who are not obese. Moving to panel B, the first two columns reveal the results of individuals who are not diagnosed with diabetes. All results are consistent with baseline model.

**Table 10:** Robustness checks.

Panel A	DBP	SBP	DBP	SBP
Sedentary*D_it_	0.022^***^	0.009	0.010^***^	−0.001
	(0.006)	(0.008)	(0.004)	(0.006)
Constant	72.806^***^	103.622^***^	80.021^***^	93.618^***^
	(19.764)	(26.251)	(13.177)	(18.672)
Obs.	311	311	519	519
*R*^2^	0.791	0.849	0.801	0.844
Panel B	DBP	SBP	DBP	SBP
Sedentary*D_it_	0.011^***^	−0.006	0.011^**^	−0.006
	(0.004)	(0.006)	(0.004)	(0.007)
Constant	69.251^***^	101.622^***^	65.494^***^	93.767^***^
	(12.315)	(18.306)	(13.162)	(20.098)
Obs.	581	581	531	531
*R*^2^	0.810	0.841	0.816	0.839

All models include fixed effects for individual, household, and year, plus all covariates.

The first two columns of Panel A are results for individuals living in the excluded provinces and those with a preference for non-salty food.

The last two columns of panel A are results for individuals who are not obese.

The first two columns of Panel B are results for individuals who are not diagnosed with diabetes.

The last two columns of panel B are results using the average DBP and SBP in last two measurements.

### 6.2 Alternative measurement of blood pressure

We also use the average DBP and SBP in last two measurements instead of the three we have at hand. The results are shown in the last two columns of Panel B of Table 10, which in line with our baseline results.

## 7 Discussion

As baby boomers born in the 1950s and 1960s reach retirement age and leave the labor market, the healthcare system faces significant challenges. Elevated blood pressure is a leading cause of mortality and disease burden worldwide (85), especially among the older adults. Meanwhile, older adults, a vulnerable group, tend to spend more time sitting daily. Leading health authorities, including the World Health Organization, emphasize the importance of reducing sedentary behavior (86). The 2018 Physical Activity Guidelines Advisory Committee graded the evidence linking sedentary behavior with mortality and cardiovascular disease as strong (87) and included the recommendation to “sit less and move more” in the 2018 federal Physical Activity Guidelines (88).

This paper utilizes the statutory retirement policy as an exogenous variation and employs the continuous difference-in-differences (DID) framework, propensity score matching difference-in-differences (PSM-DID) approach and instrumental variable (IV) method to explore the relationship between post-retirement leisure sedentary time and elevated blood pressure. Our analysis yielded two main findings. First, we observed an increase in diastolic blood pressure after retirement due to increased leisure sedentary time, while no significant increase in systolic blood pressure was noted. Second, while physical activity did not mitigate the rise in blood pressure, educational attainment and having family members employed as healthcare workers appeared to reduce its negative impact.

Regarding the first finding, our results align with previous studies and support the link between increased sedentary time and higher DBP (18, 38), confirming Hypothesis 1. Previous studies have found that elevated DBP is significantly related to male sex (89–91) and unhealthy lifestyle such as sedentary behaviors (89), smoking (90), and alcohol consumption (91). In terms of magnitude, each additional hour of sedentary behavior per day results in a statistically significant but modest increase in DBP of 0.66 mmHg on average. This finding holds clinical importance, as elevated DBP is independently linked to a higher risk of heart failure and cardiovascular mortality compared to normal blood pressure (92, 93). As such, highlighting the association between prolonged leisure sedentary time and elevated DBP could help prevent these conditions. Additionally, it remains unclear, however, why significant effects were observed for DBP but not for SBP, a pattern noted in other studies (94). One study conducted between May 2019 and December 2020 found that higher sedentary behavior was linked to increased DBP and total peripheral resistance, suggesting that sedentary behavior may primarily affect vascular stress reactivity rather than cardiac stress (18).

To better inform health interventions, our study revealed two socioeconomic mechanisms. The first mechanism found that engaging in physical activities is unlikely to alleviate the increase in blood pressure (Hypothesis 2a), aligning with literature highlighting the distinction between inactivity and sedentary behavior (44, 45). Therefore, reducing sedentary behaviors, rather than merely promoting physical activities, may be more effective in counteracting the negative impact on blood pressure. The second mechanism hypothesized that educational attainment and having family members employed as medical workers might alleviate the negative impact on blood pressure (Hypothesis 2b). This aligns with studies showing that SES is inversely related to blood pressure (95) and underscores the importance of the social gradient in health (96).

Due to data limitations, we did not explore biological pathways in detail. However, previous studies have proposed several pathways. One suggests that television viewing, often linked to unhealthy eating habits, can lead to obesity and diabetes, which increase hypertension risk (97). Another pathway relates to the suppression of skeletal muscle lipoprotein lipase (LPL) activity during prolonged sitting, which can lead to elevated levels of glucose, triglycerides, and free fatty acids. This can trigger inflammation, endothelial dysfunction, and increased sympathetic activity, potentially raising blood pressure over time (98, 99). Finally, a smaller strand of the literature has suggested that leisure sedentary time may increase DBP through pathways such as increased sympathetic activity, vagal withdrawal, and vascular resistance (34, 100, 101).

Our findings suggest three policy implications. First, while physical activity alone may not counteract the negative effects of sedentary behavior, several studies show that reducing or interrupting prolonged sitting time can lower both DBP and SBP (102). For example, short breaks every 30 min have been shown to improve blood pressure (35). A 12-month workplace-based intervention targeting Australian state government workers significantly reduced SBP by 1.0 to 3.4 mmHg (*p* < 0.01) during the first 9 months and DBP by 4–5 mmHg over the full 12 months (*p* < 0.01) (103). In practice, the Chinese government held its highest-level national health conference and subsequently announced the Outline of the Healthy China 2030 Plan in 2016, including the aim of reducing sedentary. Effective promotion of this policy could contribute to achieving these goals. Additionally, local governments should also provide affordable fitness equipment to encourage reduced sedentary time and increased physical activity. For instance, the construction of urban greenways has been shown to significantly reduce sedentary time among participants (104). Second, as habits persist post-retirement, it is crucial to help individuals establish good habits before and after retirement. This includes ensuring easy access to and understanding of health information to promote a healthy lifestyle and providing more convenient community medical services. For example, an educational intervention targeting close-to-retirement employees, including health, leisure time, and financial needs, have showed that feelings of helplessness and failure and oldness and idleness significantly decreased after the educational intervention, and feelings of effort and a new direction significantly increased (105). Third, with rapid development, urbanization, and technological advancements have led to a significant increase in sedentary behavior. According to a 2021 report, Chinese employees have an average daily sitting time of 9.4 h, with 73.9% engaging in more than 8 h sitting per day. Therefore, policymakers should consider the broader social and economic costs associated with sedentary behavior and its impact on blood pressure.

However, our study has limitations. We relied on self-reported sedentary time, which may underestimate actual sedentary behavior. Although self-reported measures are commonly used and show acceptable validity, future studies should combine them with accelerometer-assessed data for a more comprehensive assessment. Additionally, due to China’s unique retirement policy, our study focuses only on males in the formal sector. Future research should include other demographic groups to provide a broader perspective.

## Data Availability

The original contributions presented in the study are included in the article/supplementary material, further inquiries can be directed to the corresponding author.

## References

[1] Roth GA Mensah GA Johnson CO Addolorato G Ammirati E Baddour LM. Global burden of cardiovascular diseases and risk factors, 1990-2019: update from the GBD 2019 study. J Am Coll Cardiol. (2020) 76:2982–3021. doi: 10.1016/j.jacc.2020.11.010, PMID: 33309175 PMC7755038

[2] Lu J Lu Y Wang X Li X Linderman GC Wu C. Prevalence, awareness, treatment, and control of hypertension in China: data from 1·7 million adults in a population-based screening study (China PEACE million persons project). Lancet. (2017) 390:2549–58. doi: 10.1016/S0140-6736(17)32478-9 29102084

[3] Wang Z Chen Z Zhang L Wang X Hao G Zhang Z. Status of hypertension in China: results from the China hypertension survey, 2012-2015. Circulation. (2018) 137:2344–56. doi: 10.1161/CIRCULATIONAHA.117.032380 29449338

[4] Buford TW. Hypertension and aging. Ageing Res Rev. (2016) 26:96–111. doi: 10.1016/j.arr.2016.01.007 26835847 PMC4768730

[5] King DE Mainous AG 3rd Geesey ME. Turning back the clock: adopting a healthy lifestyle in middle age. Am J Med. (2007) 120:598–603. doi: 10.1016/j.amjmed.2006.09.020 17602933

[6] Yan Z Xiang N Meng J Liang H Yue Z. Understanding the effect of retirement on health behaviors in China: causality, heterogeneity and time-varying effect. Front Public Health. (2022) 10:952072. doi: 10.3389/fpubh.2022.952072, PMID: 36045724 PMC9421064

[7] Gorry D Slavov SN. The effect of retirement on health behaviors. Health Econ. (2023) 32:2234–59. doi: 10.1002/hec.4723 37340536

[8] Aldoori MI Rahman SH. Smoking and stroke: a causative role. Heavy smokers with hypertension benefit most from stopping. BMJ. (1998) 317:962–3. doi: 10.1136/bmj.317.7164.962, PMID: 9765161 PMC1114040

[9] Rehm J Anderson P Prieto JAA Armstrong I Aubin HJ Bachmann M. Towards new recommendations to reduce the burden of alcohol-induced hypertension in the European Union. BMC Med. (2017) 15:173. doi: 10.1186/s12916-017-0934-1 28954635 PMC5618725

[10] Marques FZ Nelson E Chu PY Horlock D Fiedler A Ziemann M. High-fiber diet and acetate supplementation change the gut microbiota and prevent the development of hypertension and heart failure in hypertensive mice. Circulation. (2017) 135:964–77. doi: 10.1161/CIRCULATIONAHA.116.024545 27927713

[11] Dunstan DW Howard B Healy GN Owen N. Too much sitting--a health hazard. Diabetes Res Clin Pract. (2012) 97:368–76. doi: 10.1016/j.diabres.2012.05.020 22682948

[12] Diaz KM Howard VJ Hutto B Colabianchi N Vena JE Safford MM. Patterns of sedentary behavior and mortality in U.S. middle-aged and older adults: a national cohort study. Ann Intern Med. (2017) 167:465–75. doi: 10.7326/M17-0212, PMID: 28892811 PMC5961729

[13] Harvey JA Chastin SF Skelton DA. Prevalence of sedentary behavior in older adults: a systematic review. Int J Environ Res Public Health. (2013) 10:6645–61. doi: 10.3390/ijerph10126645, PMID: 24317382 PMC3881132

[14] Van Dyck D Cardon G De Bourdeaudhuij I. Longitudinal changes in physical activity and sedentary time in adults around retirement age: what is the moderating role of retirement status, gender and educational level? BMC Public Health. (2016) 16:1125. doi: 10.1186/s12889-016-3792-4 27793134 PMC5084354

[15] van der Ploeg HP Hillsdon M. Is sedentary behaviour just physical inactivity by another name? Int J Behav Nutr Phys Act. (2017) 14:142. doi: 10.1186/s12966-017-0601-0, PMID: 29058587 PMC5651642

[16] Beunza JJ Martínez-González MA Ebrahim S Bes-Rastrollo M Núñez J Martínez JA. Sedentary behaviors and the risk of incident hypertension: the SUN cohort. Am J Hypertens. (2007) 20:1156–62. doi: 10.1016/j.amjhyper.2007.06.007 17954361

[17] Guo C Zhou Q Zhang D Qin P Li Q Tian G. Association of total sedentary behaviour and television viewing with risk of overweight/obesity, type 2 diabetes and hypertension: a dose-response meta-analysis. Diabetes Obes Metab. (2020) 22:79–90. doi: 10.1111/dom.13867 31468597

[18] Chauntry AJ Bishop NC Hamer M Kingsnorth AP Chen YL Paine NJ. Sedentary behaviour is associated with heightened cardiovascular, inflammatory and cortisol reactivity to acute psychological stress. Psychoneuroendocrinology. (2022) 141:105756. doi: 10.1016/j.psyneuen.2022.105756 35483244

[19] Hare D. Examining the timing of women's retirement in urban China: a discrete time hazard rate approach. Contemp Econ Policy. (2018) 36:451–66. doi: 10.1111/coep.12269, PMID: 30116101 PMC6089536

[20] Chen Q Deng T Bai J He X. Understanding the retirement-consumption puzzle through the lens of food consumption-fuzzy regression-discontinuity evidence from urban China. Food Policy. (2017) 73:45–61. doi: 10.1016/j.foodpol.2017.09.006

[21] Yi C Xin L. Retirement and health: evidence from China. China Econ Rev. (2018) 49:84–95. doi: 10.1016/j.chieco.2018.01.005

[22] Zhang A. Zhang Y and Tao Y does retirement make people happier? Evidence from China. Front Public Health. (2022) 10:874500. doi: 10.3389/fpubh.2022.874500 35784195 PMC9247314

[23] Wu Y Pang J Yang H. Effect of retirement on medical reimbursement expenses—evidence from China. Health Econ Rev. (2023) 13:22. doi: 10.1186/s13561-023-00434-x 37052741 PMC10099839

[24] Li H Shi X Binzhen W. The retirement consumption puzzle revisited: evidence from the mandatory retirement policy in China. J Comp Econ. (2016) 44:623–37. doi: 10.1016/j.jce.2015.06.001

[25] Li YH Ye JT. Successful retirement transition process of the elders: an empirical study in Taiwan. J Educ Stud. (2013) 9:78–91. doi: 10.14082/j.cnki.1673-1298.2013.01.004

[26] Vigezzi GP Gaetti G Gianfredi V Frascella B Gentile L Errico A. Transition to retirement impact on health and lifestyle habits: analysis from a nationwide Italian cohort. BMC Public Health. (2021) 21:1670–10. doi: 10.1186/s12889-021-11670-3, PMID: 34521363 PMC8439097

[27] Atchley RC. Retirement and leisure participation: continuity or crisis? Gerontologist. (1971) 11:13–7. doi: 10.1093/geront/11.1_Part_1.13 5579223

[28] Touvier M Bertrais S Charreire H Vergnaud AC Hercberg S Oppert JM. Changes in leisure-time physical activity and sedentary behaviour at retirement: a prospective study in middle-aged French subjects. Int J Behav Nutr Phys Act. (2010) 7:14. doi: 10.1186/1479-5868-7-14, PMID: 20181088 PMC2834610

[29] Sprod J Ferrar K Olds T Maher C. Changes in sedentary behaviours across the retirement transition: a systematic review. Age Ageing. (2015) 44:918–25. doi: 10.1093/ageing/afv140, PMID: 26504115

[30] Leskinen T Pulakka A Heinonen OJ Pentti J Kivimäki M Vahtera J. Changes in non-occupational sedentary behaviours across the retirement transition: the Finnish retirement and aging (FIREA) study. J Epidemiol Community Health. (2018) 72:695–701. doi: 10.1136/jech-2017-209958, PMID: 29636399

[31] Suorsa K Pulakka A Leskinen T Pentti J Vahtera J Stenholm S. Changes in prolonged sedentary behaviour across the transition to retirement. Occup Environ Med. (2020) 78:409–12. doi: 10.1136/oemed-2020-106532, PMID: 33203649 PMC8142433

[32] Bossé R Ekerdt DJ. Change in self-perception of leisure activities with retirement. Gerontologist. (1981) 21:650–4. doi: 10.1093/geront/21.6.650 7333492

[33] Evenson KR Rosamond WD Cai J Diez-Roux AV Brancati FL Atherosclerosis Risk in Communities Study Investigators. Influence of retirement on leisure-time physical activity: the atherosclerosis risk in communities study. Am J Epidemiol. (2002) 155:692–9. doi: 10.1093/aje/155.8.692 11943686

[34] Dempsey PC Larsen RN Dunstan DW Owen N Kingwell BA. Sitting less and moving more: implications for hypertension. Hypertension. (2018) 72:1037–46. doi: 10.1161/HYPERTENSIONAHA.118.11190, PMID: 30354827 PMC7343526

[35] Ravichandran S Sukumar S Chandrasekaran B Kadavigere R N SK Palaniswamy HP. Influence of sedentary behaviour interventions on vascular functions and cognitive functions in hypertensive adults-a scoping review on potential mechanisms and recommendations. Int J Environ Res Public Health. (2022) 19:15120. doi: 10.3390/ijerph192215120, PMID: 36429835 PMC9690278

[36] Lim MS Park B Kong IG Sim S Kim SY Kim JH. Leisure sedentary time is differentially associated with hypertension, diabetes mellitus, and hyperlipidemia depending on occupation. BMC Public Health. (2017) 17:278. doi: 10.1186/s12889-017-4192-0, PMID: 28335768 PMC5364658

[37] Li Z Zhong W Gao J Zhang X Lin G Qi C. Association between leisure sedentary behaviors and hypertension risk: a prospective cohort study and two-sample Mendelian randomization analysis in Europeans. Prev Med. (2024) 181:107915. doi: 10.1016/j.ypmed.2024.107915, PMID: 38408649

[38] Gallagher J Carr LJ. Leisure but not occupational physical activity and sedentary behavior associated with better health. J Occup Environ Med. (2021) 63:e774–82. doi: 10.1097/JOM.0000000000002365, PMID: 34456325

[39] Hashem R Rey-Lόpez JP Hamer M McMunn A Rowlands A Whincup PH. Associations between objectively assessed and questionnaire-based sedentary behaviour with body mass index and systolic blood pressure in Kuwaiti adolescents. BMC Res Notes. (2019) 12:588. doi: 10.1186/s13104-019-4626-0, PMID: 31533859 PMC6751576

[40] Almoosawi S Winter J Prynne CJ Hardy R Stephen AM. Daily profiles of energy and nutrient intakes: are eating profiles changing over time? Eur J Clin Nutr. (2012) 66:678–86. doi: 10.1038/ejcn.2011.210, PMID: 22190135 PMC3389619

[41] Dunstan DW Kingwell BA Larsen R Healy GN Cerin E Hamilton MT. Breaking up prolonged sitting reduces postprandial glucose and insulin responses. Diabetes Care. (2012) 35:976–83. doi: 10.2337/dc11-1931 22374636 PMC3329818

[42] Carter S Hartman Y Holder S Thijssen DH Hopkins ND. Sedentary behavior and cardiovascular disease risk: mediating mechanisms. Exerc Sport Sci Rev. (2017) 45:80–6. doi: 10.1249/JES.0000000000000106, PMID: 28118158

[43] Wang Q. Changes in blood pressure during the transition of retirement: the role of physical activity in China. J Hum Hypertens. (2020) 34:536–43. doi: 10.1038/s41371-019-0277-9 31664173

[44] Sedentary Behaviour Research Network. Letter to the editor: standardized use of the terms “sedentary” and “sedentary behaviours”. Appl Physiol Nutr Metab. (2012) 37:540–2. doi: 10.1139/h2012-024 22540258

[45] Tremblay MS Aubert S Barnes JD Saunders TJ Carson V Latimer-Cheung AE. Sedentary behavior research network (SBRN) - terminology consensus project process and outcome. Int J Behav Nutr Phys Act. (2017) 14:75. doi: 10.1186/s12966-017-0525-8, PMID: 28599680 PMC5466781

[46] Adler NE Newman K. Socioeconomic disparities in health: pathways and policies. Health Aff (Millwood). (2002) 21:60–76. doi: 10.1377/hlthaff.21.2.60 11900187

[47] Li J Powdthavee N. Does more education lead to better health habits? Evidence from the school reforms in Australia. Soc Sci Med. (2015) 127:83–91. doi: 10.1016/j.socscimed.2014.07.021 25028347

[48] Sumimoto Y Yanagita M Miyamatsu N Okuda N Nishi N Nakamura Y. Association between socioeconomic status and prolonged television viewing time in a general Japanese population: NIPPON DATA2010. Environ Health Prev Med. (2021) 26:57. doi: 10.1186/s12199-021-00978-6, PMID: 33962567 PMC8105981

[49] Moga Rogoz AT Sart G Bayar Y Gavriletea MD. Impact of economic freedom and educational attainment on life expectancy: evidence from the new EU member states. Front Public Health. (2022) 10:907138. doi: 10.3389/fpubh.2022.907138, PMID: 35844897 PMC9280055

[50] Apfel F Tsouros AD. Health literacy: the solid facts. Copenhagen: World Health Organization. (2013) 3–26.

[51] Wolf MS Gazmararian JA Baker DW. Health literacy and health risk behaviors among older adults. Am J Prev Med. (2007) 32:19–24. doi: 10.1016/j.amepre.2006.08.024 17184964

[52] McNaughton CD Kripalani S Cawthon C Mion LC Wallston KA Roumie CL. Association of health literacy with elevated blood pressure: a cohort study of hospitalized patients. Med Care. (2014) 52:346–53. doi: 10.1097/MLR.0000000000000101 24556896 PMC4031281

[53] Zhao M Konishi Y Glewwe P. Does information on health status lead to a healthier lifestyle? Evidence from China on the effect of hypertension diagnosis on food consumption. J Health Econ. (2013) 32:367–85. doi: 10.1016/j.jhealeco.2012.11.007, PMID: 23334058

[54] Ciancio A Kämpfen F Kohler HP Kohler IV. Health screening for emerging non-communicable disease burdens among the global poor: evidence from sub-Saharan Africa. J Health Econ. (2021) 75:102388. doi: 10.1016/j.jhealeco.2020.102388, PMID: 33249266 PMC7855787

[55] Lubans DR Hesketh K Cliff DP Barnett LM Salmon J Dollman J. A systematic review of the validity and reliability of sedentary behaviour measures used with children and adolescents. Obes Rev. (2011) 12:781–99. doi: 10.1111/j.1467-789X.2011.00896.x 21676153

[56] Su Y Li X Li H Xu J Xiang M. Association between sedentary behavior during leisure time and excessive weight in Chinese children, adolescents, and adults. Nutrients. (2023) 15:424. doi: 10.3390/nu15020424 36678295 PMC9867297

[57] Cao B Zhao Y Ren Z McIntyre RS Teopiz KM Gao X. Are physical activities associated with perceived stress? The evidence from the China health and nutrition survey. Front Public Health. (2021) 9:697484. doi: 10.3389/fpubh.2021.697484, PMID: 34414158 PMC8369204

[58] Zhou C Wu Q Ye Z Liu M Zhang Z Zhang Y. Inverse association between variety of proteins with appropriate quantity from different food sources and new-onset hypertension. Hypertension. (2022) 79:1017–27. doi: 10.1161/HYPERTENSIONAHA.121.18222, PMID: 35264000

[59] Schaare HL Blöchl M Kumral D Uhlig M Lemcke L Valk SL. Associations between mental health, blood pressure and the development of hypertension. Nat Commun. (2023) 14:1953. doi: 10.1038/s41467-023-37579-6, PMID: 37029103 PMC10082210

[60] Wang Z Yang T Fu H. Prevalence of diabetes and hypertension and their interaction effects on cardio-cerebrovascular diseases: a cross-sectional study. BMC Public Health. (2021) 21:1224. doi: 10.1186/s12889-021-11122-y 34172039 PMC8229421

[61] Chen F Zhang X Chen Z. Air pollution and mental health: evidence from China health and nutrition survey. J Asian Econ. (2023) 86:101611. doi: 10.1016/j.asieco.2023.101611

[62] Shahab H Khan HS Almas A Khan SA Artani A Khan AH. Defining the hemodynamic response of hypertensive and normotensive subjects through serial timed blood pressure readings in the clinic. Clin Hypertens. (2019) 25:8. doi: 10.1186/s40885-019-0114-z, PMID: 30984413 PMC6442419

[63] Waghorn J Liu H Yanlin W Rayner SE Kimmerly DS O’Brien MW. A single bout of prolonged sitting augments very short-term blood pressure variability. Am J Hypertens. (2024) 37:700–7. doi: 10.1093/ajh/hpae055 38703068 PMC11322278

[64] Plotnikoff R Karunamuni N. Reducing sitting time: the new workplace health priority. Arch Environ Occup Health. (2012) 67:125–7. doi: 10.1080/19338244.2012.697407 22845724

[65] Krtalic B Knezevic T Zeljkovic-Vrkic T Kos J Pecin I Gellineo L. Family history, blood pressure and life style. Result from EHUH study. J Hypertens. (2019) 37:e230–17. doi: 10.1097/01.hjh.0000572956.60503.d4

[66] Zhao J Zuo L Sun J Su C Wang H. Geographic and urban–rural disparities in dietary energy and macronutrient composition among women of childbearing age: findings from the China health and nutrition survey, 1991–2015. Nutr J. (2023) 22:23. doi: 10.1186/s12937-023-00851-y, PMID: 37158933 PMC10169383

[67] Burt VL Whelton P Roccella EJ Brown C Cutler JA Higgins M. Prevalence of hypertension in the US adult population. Results from the third national health and nutrition examination survey, 1988-1991. Hypertension. (1995) 25:305–13. doi: 10.1161/01.hyp.25.3.305 7875754

[68] Zhang Y Salm M van Soest A. The effect of retirement on healthcare utilization: evidence from China. J Health Econ. (2018) 62:165–77. doi: 10.1016/j.jhealeco.2018.09.009 30390499

[69] Hou B Wang G Wang Y Zhao Y. The health capacity to work at older ages in urban China. China Econ Rev. (2021) 66:101581. doi: 10.1016/j.chieco.2020.101581, PMID: 35444378 PMC9017986

[70] Liu DT-W. The effects of institutionalization in China: a difference-in-differences analysis of the mandatory retirement age. China Econ Rev. (2018) 52:192–203. doi: 10.1016/j.chieco.2018.07.005

[71] Rosenbaum PR Rubin DB. The central role of the propensity score in observational studies for causal effects. Biometrika. (1983) 70:41–55. doi: 10.1093/biomet/70.1.41

[72] Copeland JL Ashe MC Biddle SJH Brown WJ Buman MP Chastin S. Sedentary time in older adults: a critical review of measurement, associations with health, and interventions. Br J Sports Med. (2017) 51:1539. doi: 10.1136/bjsports-2016-097210, PMID: 28724714

[73] Lewbel A. Constructing instruments for regressions with measurement error when no additional data are available, with an application to patents and R&D. Econometrica. (1997) 65:1201–13. doi: 10.2307/2171884

[74] Hu BY Johnson GK Wu H. Screen time relationship of Chinese parents and their children. Child Youth Serv Rev. (2018) 94:659–69. doi: 10.1016/j.childyouth.2018.09.008

[75] Munasib A Bhattacharya S. Is the ‘Idiot's box’ raising idiocy? Early and middle childhood television watching and child cognitive outcome. Econ Educ Rev. (2010) 29:873–83. doi: 10.1016/j.econedurev.2010.03.005

[76] Caspersen CJ Powell KE Christenson GM. Physical activity, exercise, and physical fitness: definitions and distinctions for health-related research. Public Health Rep. (1985) 100:126–31. 3920711 PMC1424733

[77] U.S. Department of Health and Human Services, Public Health Service, Centers for Disease Control and Prevention, National Center for Chronic Disease Prevention and Health Promotion, Division of Nutrition and Physical Activity. Promoting physical activity: a guide for community action. Champaign: Human Kinetics (1999).

[78] Adler NE Rehkopf DH. U.S. disparities in health: descriptions, causes, and mechanisms. Annu Rev Public Health. (2008) 29:235–52. doi: 10.1146/annurev.publhealth.29.020907.090852 18031225

[79] Lee PH Wong FK. The association between time spent in sedentary behaviors and blood pressure: a systematic review and meta-analysis. Sports Med. (2015) 45:867–80. doi: 10.1007/s40279-015-0322-y, PMID: 25749843

[80] Stock JH Yogo M. Testing for weak instruments in linear IV regression. NBER Tech Work Pap. (2005) 14:80–108. doi: 10.1017/CBO9780511614491.006

[81] Acemoglu D Johnson S Robinson JA. The colonial origins of comparative development: an empirical investigation. Am Econ Rev. (2001) 91:1369–401. doi: 10.1257/aer.91.5.1369

[82] Thorp AA Owen N Neuhaus M Dunstan DW. Sedentary behaviors and subsequent health outcomes in adults a systematic review of longitudinal studies, 1996-2011. Am J Prev Med. (2011) 41:207–15. doi: 10.1016/j.amepre.2011.05.004 21767729

[83] Link BG Phelan J. Social conditions as fundamental causes of disease. J Health Soc Behav. (1995) 35:80. doi: 10.2307/2626958 7560851

[84] Yin M Augustin B Fu Z Yan M Fu A Yin P. Geographic distributions in hypertension diagnosis, measurement, prevalence, awareness, treatment and control rates among middle-aged and older adults in China. Sci Rep. (2016) 6:37020. doi: 10.1038/srep37020, PMID: 27841326 PMC5107929

[85] Zhou B Perel P Mensah GA Ezzati M. Global epidemiology, health burden and effective interventions for elevated blood pressure and hypertension. Nat Rev Cardiol. (2021) 18:785–802. doi: 10.1038/s41569-021-00559-8, PMID: 34050340 PMC8162166

[86] Bull FC al-Ansari SS Biddle S Borodulin K Buman MP Cardon G. World Health Organization 2020 guidelines on physical activity and sedentary behavior. Br J Sports Med. (2020) 54:1451–62. doi: 10.1136/bjsports-2020-102955, PMID: 33239350 PMC7719906

[87] Katzmarzyk PT Powell KE Jakicic JM Troiano RP Piercy K Tennant B. Sedentary behavior and health: update from the 2018 physical activity guidelines advisory committee. Med Sci Sports Exerc. (2019) 51:1227–41. doi: 10.1249/MSS.0000000000001935, PMID: 31095080 PMC6527341

[88] Piercy KL Troiano RP. Physical activity guidelines for Americans from the US Department of Health and Human Services. Circ Cardiovasc Qual Outcomes. (2018) 11:e005263. doi: 10.1161/CIRCOUTCOMES.118.005263 30571339

[89] Midha T Lalchandani A Nath B Kumari R Pandey U. Prevalence of isolated diastolic hypertension and associated risk factors among adults in Kanpur, India. Indian Heart J. (2012) 64:374–9. doi: 10.1016/j.ihj.2012.06.007 22929820 PMC3860613

[90] Saeed AA Al-Hamdan NA. Isolated diastolic hypertension among adults in Saudi Arabia: prevalence, risk factors, predictors and treatment. Results of a National Survey. Balkan Med J. (2016) 33:52–7. doi: 10.5152/balkanmedj.2015.153022, PMID: 26966618 PMC4767310

[91] Mahajan S Zhang D He S Lu Y Gupta A Spatz ES. Prevalence, awareness, and treatment of isolated diastolic hypertension: insights from the China PEACE million persons project. J Am Heart Assoc. (2019) 8:e012954. doi: 10.1161/JAHA.119.012954, PMID: 31566101 PMC6806046

[92] Sheriff HM Tsimploulis A Valentova M Anker MS Deedwania P Banach M. Isolated diastolic hypertension and incident heart failure in community-dwelling older adults: insights from the cardiovascular health study. Int J Cardiol. (2017) 238:140–3. doi: 10.1016/j.ijcard.2017.02.142, PMID: 28343761 PMC6454920

[93] Arima H Anderson C Omae T Woodward M Hata J Murakami Y. Effects of blood pressure lowering on major vascular events among patients with isolated diastolic hypertension: the perindopril protection against recurrent stroke study (PROGRESS) trial. Stroke. (2011) 42:2339–41. doi: 10.1161/STROKEAHA.110.606764 21700945

[94] Edwardson CL Henson J Biddle SJH Davies MJ Khunti K Maylor B. activPAL and ActiGraph assessed sedentary behavior and cardiometabolic health markers. Med Sci Sports Exerc. (2020) 52:391–7. doi: 10.1249/MSS.0000000000002138 31479008

[95] Shen A Chen C Zhang Z Zhou J Lv Y Wang J. Associations between socioeconomic status and rates of blood pressure changes among Chinese older adults: a longitudinal community-based cohort study. Public Health. (2024) 232:121–7. doi: 10.1016/j.puhe.2024.04.027 38772200

[96] Marmot M Bell R. Fair society, healthy lives. Public Health. (2012) 126:S4–S10. doi: 10.1016/j.puhe.2012.05.014 22784581

[97] Schaan CW Cureau FV Salvo D Kohl HW 3rd Schaan BD. Unhealthy snack intake modifies the association between screen-based sedentary time and metabolic syndrome in Brazilian adolescents. Int J Behav Nutr Phys Act. (2019) 16:115. doi: 10.1186/s12966-019-0880-8, PMID: 31775773 PMC6882160

[98] Hamilton MT Hamilton DG Zderic TW. Exercise physiology versus inactivity physiology: an essential concept for understanding lipoprotein lipase regulation. Exerc Sport Sci Rev. (2004) 32:161–6. doi: 10.1097/00003677-200410000-00007, PMID: 15604935 PMC4312662

[99] Hamilton MT Hamilton DG Zderic TW. Role of low energy expenditure and sitting in obesity, metabolic syndrome, type 2 diabetes, and cardiovascular disease. Diabetes. (2007) 56:2655–67. doi: 10.2337/db07-0882, PMID: 17827399

[100] Brindle RC Ginty AT Phillips AC Carroll D. A tale of two mechanisms: a meta-analytic approach toward understanding the autonomic basis of cardiovascular reactivity to acute psychological stress. Psychophysiology. (2014) 51:964–76. doi: 10.1111/psyp.12248, PMID: 24924500

[101] Ferreira-Silva R Goya TT Barbosa ERF Durante BG Araujo CEL Lorenzi-Filho G. Vascular response during mental stress in sedentary and physically active patients with obstructive sleep apnea. J Clin Sleep Med. (2018) 14:1463–70. doi: 10.5664/jcsm.7314, PMID: 30176967 PMC6134250

[102] Larsen RN Kingwell BA Sethi P Cerin E Owen N Dunstan DW. Breaking up prolonged sitting reduces resting blood pressure in overweight/obese adults. Nutr Metab Cardiovasc Dis. (2014) 24:976–82. doi: 10.1016/j.numecd.2014.04.011 24875670

[103] Mainsbridge C Ahuja K Williams A Bird ML Cooley D Pedersen SJ. Blood pressure response to interrupting workplace sitting time with non-exercise physical activity: results of a 12-month cohort study. J Occup Environ Med. (2018) 60:769–74. doi: 10.1097/JOM.0000000000001377, PMID: 29905645 PMC6125747

[104] Li Z Lu Y Xie B Wu Y. Large-scale greenway exposure reduces sedentary behavior: a natural experiment in China. Health Place. (2024) 89:103283. doi: 10.1016/j.healthplace.2024.103283, PMID: 38850725

[105] Najafipour H Sepehri GH Saberi SH Kashef H Borhaninejad V. Design and evaluation of an educational intervention to prepare close-to-retirement employees for retirement. Adv Gerontol. (2022) 12:162–7. doi: 10.1134/S2079057022020138

